# A Rare Case of Cystic Fibrosis Transmembrane Conductance Regulator (CFTR) Gene Mutation on Exon 8 in a Patient Presenting With Recurrent Infections and Failure to Thrive

**DOI:** 10.7759/cureus.67892

**Published:** 2024-08-27

**Authors:** Sudhir Malwade, Ruhi Shaligram, Balakrushna P Garud, Shailaja Mane

**Affiliations:** 1 Pediatrics, Dr. D.Y. Patil Medical College, Hospital and Research Center, Dr. D. Y. Patil Vidyapeeth (Deemed to be University) Pimpri, Pune, IND

**Keywords:** recurrent infections, cftr mutation, failure-to-thrive, genetic disorder, cystic fibrosis

## Abstract

Cystic fibrosis (CF) is a genetic disorder that affects various bodily organs, predominantly the pulmonary and gastrointestinal systems. Identifying CF at an early stage can pose a significant challenge, especially when symptoms manifest unusually. The following case study depicts an exceptional and atypical instance of CF in a neonate. A male infant aged 4 months exhibited symptoms such as failure to thrive (FTT), inadequate weight gain, feeding difficulties, slight developmental delay (presence of head lag), and sporadic irritability. The patient experienced an uncomplicated prenatal and postnatal period. Subsequently, the patient suffered from recurring infections and a notable inability to gain weight. Initial tests, encompassing assessments of liver functionality and metabolic processes, yielded inconclusive results. A genetic assessment pinpointed a detrimental cystic fibrosis transmembrane conductance regulator (*CFTR*) gene mutation on Exon 8, thereby confirming the presence of CF. This analysis underscores the importance of considering CF even in the absence of typical indications. Timely and precise identification through genetic analysis is imperative for effective treatment and enhanced prognoses among individuals with CF.

## Introduction

Cystic fibrosis (CF) is a genetic disorder caused by mutations in the cystic fibrosis transmembrane conductance regulator (*CFTR*) gene [1]. This gene codes for a protein that is involved in chloride and sodium transport across epithelial membranes. This condition leads to the production of thick, sticky mucus that can lead to blockages and infections The lungs, pancreas, and gastrointestinal system are the most commonly seen in this condition [1]. CF is a common genetic disorder among populations of European descent but varies significantly worldwide. The incidence of CF in populations of European origin is estimated to be approximately 1 in 2,500 to 1 in 3,500 live births [2] and a similar incidence has been found in the populations of North America, and Australia [3]. However, the incidence of CF is much lower in populations of Asian and African descent and hence requires a higher index of suspicion for accurate diagnosis. For instance, in Japan, the incidence is about 1 in 350,000 live births, and in African populations, the incidence is approximately 1 in 15,000 to 1 in 20,000 live births [4].

In India, CF is considered rare, although it is likely underdiagnosed due to limited awareness and diagnostic facilities. The incidence is reported as 1 in 10,000 to 1 in 40,000 live births [5]. Studies and reports suggest that CF is underreported, and the actual incidence might be higher, reflecting a need for better diagnostic practices and awareness among healthcare providers in the country [6]

The incidence of specific *CFTR* gene mutations, including those on exon 8, varies globally and within different populations. The *CFTR* gene mutation c.1029del (p.Cys343Ter) on exon 8 is one of the many mutations that can cause CF, but its frequency compared to more common mutations like ΔF508 is much lower. The most common *CFTR *mutation globally is ΔF508, which accounts for about 70% of CF cases in Caucasians [7]. Mutations on exon 8, such as c.1029del (p.Cys343Ter), are much less common. The overall frequency of such mutations varies, but they are considered rare compared to ΔF508. Comprehensive studies and CF mutation databases like the CFTR2 project have cataloged over 2,000 different *CFTR *mutations, but detailed prevalence data for many of these, including c.1029del, is often limited. This mutation in the *CFTR *gene is a frameshift type of mutation. In the European population, the frequency of mutations on exon 8 is relatively low and varies across different countries and ethnic groups [8].

In this report, we present a rare case of CF in a 4-month-old male who was initially referred for evaluation of failure to thrive (FTT), pneumonia, and recurrent urinary tract infections (UTIs). This highlights the diagnostic challenges and clinical management of the condition. The rarity of this case, particularly with the c.1029del (p.Cys343Ter) mutation on exon 8, underscores the importance of thorough genetic testing and awareness of CF in diverse populations.

## Case presentation

A 4-month-old male presented to our tertiary care centre with complaints of poor weight gain (below the 5th percentile for weight on growth charts), difficulty feeding, poor suck, mild developmental delay, and intermittent irritability. These symptoms were indicative of Failure to Thrive (FTT). The patient was born to a 27-year-old mother, G2P2L1, at term gestation in a non-consanguineous marriage. The pregnancy and postnatal period were uneventful. The birth weight was 3.5 kg, and no resuscitation was needed. The baby was discharged on day 4 with breastfeeding. Two days post-discharge, the patient was admitted to the NICU for exaggerated physiological jaundice. The unconjugated serum bilirubin was 21 μmol/L (reference range: 3.4-17.1 μmol/L) and there was blood group incompatibility. The haemogram was normal. The baby received phototherapy for two days and was discharged on breastfeeding.

At four months, the baby, now weighing 2.9 kg (a 17% weight loss since birth), was brought to our center with FTT. The baby was being breastfed with intermittent top-up feeds. There was no history of vomiting, regurgitation, or loose motions. Stools were well-formed, and previous stool examinations were normal. On examination, the skin was dry and pale, indicating mild dehydration. The abdomen was mildly distended, and hepatosplenomegaly was present. The liver was firm and measured 4 cm, and the spleen measured 3 cm. Liver function tests showed serum glutamic-pyruvic transaminase (SGPT) 145 U/L (Normal 7-56 U/L), serum glutamic-oxaloacetic transaminase (SGOT) 25 U/L (Normal 5-40 U/L), and alkaline phosphatase (ALP) 225 U/L (Normal 44-147 U/L).

Neurological examination revealed gross hypotonia and poor reflexes. There were no dysmorphic features or neurocutaneous markers. Developmentally, the baby could not turn on its sides. Vision and automated auditory brainstem response (AABR) were normal. Cardiovascular examination revealed a haemic murmur, but there were no signs of heart failure.

We counseled the parents on measured breastfeeding. The following week, the patient gained 150 grams, with no new complaints. However, two days later, the baby presented with a high-grade fever, cough, and coryza, along with decreased feeding. The baby was admitted, and evaluation revealed a *Klebsiella *urinary tract infection (UTI). The patient was treated for 14 days with antibiotics, but the baby's weight was reduced to 2.8 kg.

Post-discharge, within a week, the patient developed pneumonia, necessitating another admission. The patient was treated for 14 days. Given the hepatosplenomegaly, TORCH (toxoplasmosis, rubella cytomegalovirus, herpes simplex, and HIV) titers were organized during this course, which came back negative. The rest of the study was normal. Thyroid function tests were normal. A haemogram showed haemoglobin (Hb) of 6g/dL ( normal range 9.5-14 g/dL), with slight microcytosis. Iron studies were normal, leading to a bone marrow study, which was normal, concluding the patient had anemia of chronic diseases.

During this admission, the baby experienced asymptomatic hypoglycemia. In view of hepatomegaly and hypoglycemia, a metabolic workup was discussed with the parents. The patient was discharged on a measured feeding schedule.

Within a week, the patient was readmitted with high-grade fever and decreased feeding. Another *Klebsiella* UTI was detected and treated with higher antibiotics. Given the frequent readmissions, a metabolic workup including was done. tandem mass spectrometry (TMS) and gas chromatography-mass spectrometry (GCMS) reports were normal. Gross hypotonia and absent reflexes persisted, though vision and social smile were appropriate. Urine tests for ketones and reducing substances were positive. The tests were inconclusive therefore diagnosing this patient with clinical indications of severe failure to thrive, recurrent anemia, and recurrent infections required further evaluation for genetic pathogenic variations. Clinical exome sequencing revealed a pathogenic variant in the cystic fibrosis transmembrane conductance regulator (CFTR) gene (ENST00000003084.6) located on Exon 8, c.1029del (p.Cys343Ter), with a homozygous zygosity (Table 1). This mutation is associated with Cystic Fibrosis (OMIM (Online Mendelian Inheritance in Man database)), an autosomal recessive disease. The identified variant is classified as pathogenic and causative of the patient's reported phenotype

**Table 1:** Clinical exome sequencing report OMIM: Online Mendelian Inheritance in Man database

Gene (Transcript)	Location	Variant	Zygosity	Disease (OMIM)	Inheritance	Classification
CFTR(+) (ENST00000003084.6)	Exon 8	c.1029del (p.Cyd343Ter)	Homozygous	Cystic fibrosis	Autosomal recessive	Pathogenic

The genetic study report was discussed with the parents, and counseling was provided regarding the patient's condition and the need to perform a sweat chloride test. Unfortunately, before further investigations could be conducted, the patient succumbed to death due to a deterioration in clinical condition from recurrent infections. 

## Discussion

This case report discusses the unusual presentation of cystic fibrosis (CF) in a 4-month-old male having atypical clinical features. Instead of the generally expected respiratory and gastrointestinal symptoms such as chronic cough, malabsorption, and steatorrhea, the patient exhibited a failure to thrive (FTT) and recurrent infections of the urinary tract and pneumonia. The absence of classic gastrointestinal signs like greasy stools initially complicated diagnosis, emphasizing the diagnostic challenges posed by atypical presentations of CF.

In most CF cases, symptoms manifest early with respiratory and digestive issues due to mutations in the cystic fibrosis transmembrane conductance regulator (*CFTR*) gene [9]. However, this patient's presentation was characterized by hepatosplenomegaly and recurrent infections, which diverted initial clinical suspicion away from CF. Genetic testing ultimately revealed a pathogenic mutation (c.1029del, p.Cys343Ter) in the *CFTR *gene Exon 8, marking the first reported case of this specific mutation in India and highlighting its importance in the evaluation of complex cases [7, 8]. This underscores the critical role of genetic testing in confirming CF diagnoses, especially in cases where clinical symptoms do not align with classic presentations.

Upon confirmation of cystic fibrosis, the management plan included addressing the patient’s nutritional needs, treating infections, and initiating CF-specific therapies. Ensuring adequate caloric intake was prioritized, and the patient was placed on a measured feeding schedule. Despite these efforts, the patient continued to experience recurrent infections, necessitating antibiotic treatments and preventive measures to minimize future risks [10]. The presence of hepatosplenomegaly and anemia of chronic disease required regular follow-ups and comprehensive care from a multidisciplinary team. A team including pediatricians, dietitians, and genetic counselors was consulted to closely monitor the patient’s growth and development and address various complications associated with cystic fibrosis [11].

While CF is known for its variability in presentation, the lack of gastrointestinal symptoms, along with the presence of hepatosplenomegaly and recurrent urinary tract infections, was particularly misleading. In most CF cases, infants exhibit symptoms such as chronic cough, wheezing, frequent lung infections, and difficulty gaining weight due to malabsorption [12]. However, this case underscores the importance of considering CF even when classic symptoms are absent, as genetic mutation can lead to diverse clinical manifestations [13].

The diagnostic journey was prolonged due to the patient's atypical symptoms, necessitating extensive metabolic workups, imaging studies, and liver function tests before genetic testing provided clarity. Management involved tailored nutritional support, infection treatment with antibiotics, and initiation of CF-specific therapies aimed at improving the patient's overall health and growth. Despite these interventions, recurrent infections persisted, requiring ongoing medical intervention and close monitoring by a multidisciplinary team comprising pediatricians, dietitians, and genetic counselors. This holistic approach aimed to mitigate complications associated with CF, including the management of hepatosplenomegaly and chronic anemia, ensuring comprehensive care and support for the patient's complex medical needs.

## Conclusions

This case exemplifies the variability in Cystic fibrosis (CF) presentations and underscores the diagnostic challenges associated with atypical cases. The absence of classic gastrointestinal symptoms initially misled the diagnostic process, emphasizing the need for a high index of suspicion for CF in infants with unexplained failure to thrive and recurrent infections. Early and accurate diagnosis through genetic testing is essential for managing CF and improving patient outcomes. Clinicians should consider CF in the differential diagnosis of infants presenting with failure to thrive and recurrent infections, even in the absence of classic symptoms. This underscores the importance of thorough evaluation and genetic testing in suspected cases of CF to ensure timely intervention and appropriate management.

## References

[1] Dickinson KM, Collaco JM (2021). Cystic fibrosis. Pediatr Rev.

[2] Elborn JS (2016). Cystic fibrosis. Lancet.

[3] Farrell PM, White TB, Ren CL (2017). Diagnosis of cystic fibrosis: consensus guidelines from the Cystic Fibrosis Foundation. J Pediatr.

[4] (2024). Programme WHO: The molecular genetic epidemiology of cystic fibrosis : report of a joint meeting of WHO/IECFTN/ICF(M)A/ECFS, Genoa, Italy, 19 June 2002. https://iris.who.int/bitstream/handle/10665/68702/WHO_HGN_CF_WG_04.02.pdf?sequence=1&isAllowed=y.

[5] Kabra SK, Kabra M, Lodha R (2003). Clinical profile and frequency of delta f508 mutation in Indian children with cystic fibrosis. Indian Pediatr.

[6] Mandal A, Kabra S, Lodha R (2015). Cystic fibrosis in india: past, present and future. HSOA J Pulmon Med Respir Res.

[7] Cutting GR (2015). Cystic fibrosis genetics: from molecular understanding to clinical application. Nat Rev Genet.

[8] Sosnay PR, Siklosi KR, Van Goor F (2013). Defining the disease liability of variants in the cystic fibrosis transmembrane conductance regulator gene. Nat Genet.

[9] Maurya N, Awasthi S, Dixit P (2012). Association of CFTR gene mutation with bronchial asthma. Indian J Med Res.

[10] Kerem E, Reisman J, Corey M, Canny GJ, Levison H (1992). Prediction of mortality in patients with cystic fibrosis. N Engl J Med.

[11] Madde A, Okoniewski W, Sanders DB, Ren CL, Weiner DJ, Forno E (2022). Nutritional status and lung function in children with pancreatic-sufficient cystic fibrosis. J Cyst Fibros.

[12] Ratjen F, Döring G (2003). Cystic fibrosis. Lancet.

[13] De Boeck K, Wilschanski M, Castellani C, Taylor C, Cuppens H, Dodge J, Sinaasappel M (2006). Cystic fibrosis: terminology and diagnostic algorithms. Thorax.

